# Investigating the association between the number of interpersonal supporters during first-time pregnancy and postpartum depression symptoms

**DOI:** 10.1017/S2045796025000241

**Published:** 2025-06-27

**Authors:** Junko Niimura, Syudo Yamasaki, Miharu Nakanishi, Satoshi Yamaguchi, Kaori Baba, Naomi Nakajima, Mitsuhiro Miyashita, Daniel Stanyon, Gemma Knowles, Jordan DeVylder, Mariko Hiraiwa-Hasegawa, Shuntaro Ando, Kiyoto Kasai, Atsushi Nishida

**Affiliations:** 1 Mental Health Promotion Unit, Research Center for Social Science & Medicine, Tokyo Metropolitan Institute of Medical Science, Tokyo, Japan; 2 Department of Psychiatric Nursing, Tohoku University Graduate School of Medicine, Miyagi, Japan; 3 Economic and Social Research Council (ESRC) Center for Society and Mental Health, Institute of Psychiatry, Psychology, and Neuroscience, King’s College London, London, UK; 4 Silver School of Social Work, New York University, New York, USA; 5 Department of Neuropsychiatry, Graduate School of Medicine, University of Tokyo, Tokyo, Japan

**Keywords:** Cohort study, first births, interpersonal support, postpartum depression, social support, young mothers

## Abstract

**Aims:**

First-year postpartum depression is a common mental health problem among first-time mothers. A younger age of pregnancy often compounds the challenge due to underlying factors such as poverty and limited educational achievement. This study aimed to examine the minimal number of interpersonal supporters during pregnancy associated with lower levels of postpartum depressive symptoms among first-time mothers.

**Methods:**

We obtained data from the population-based Mother–Infant/Newborn Tokyo Cohort (MINT cohort) in four municipalities in Tokyo on 429 first-time mothers who responded to two waves of surveys (early pregnancy and one month postpartum). They completed self-report measures of interpersonal support using one item from the Social Support Questionnaire and depressive symptoms using the Edinburgh Postnatal Depression Scale. Segmented regression analyses were conducted to determine the threshold at which the strength of the association changed between the number of interpersonal supporters and postpartum depressive symptoms, with adjustment for depressive symptoms in pregnancy. This analysis was also conducted with the sample stratified into young mothers (≤ 25 years) and older mothers (≥ 26 years).

**Results:**

In the overall sample, postpartum depressive symptoms were found to be lower among individuals with more than 3.0 supportive individuals (prepartum). Among young mothers, this threshold was higher, with lower symptom levels observed among those with at least 5.3 supporters. Only 22.9% of young first-time mothers had this level of interpersonal support, compared to 54.8% of all first-time mothers.

**Conclusions:**

Our results suggest that having four or more interpersonal supporters in early pregnancy is associated with lower levels of postpartum depressive symptoms among first-time mothers. Additionally, among young mothers, having six or more supporters was associated with lower postpartum depressive symptoms. These findings suggest that tailored strategies to increase supporters around first-time pregnant women might be beneficial depending on their age.

## Introduction

First-time mothers constitute a priority population for public mental health strategies (Leahy-Warren *et al.*, 2012; Ong *et al.*, 2023; Wilkins, 2006). Maternal depression in the first year postpartum can have serious consequences, including maternal morbidity and mortality as well as adverse infant outcomes (Arditi-Arbel *et al.*, 2023; Howard *et al.*, 2014; Moreira *et al.*, 2023; Rogers *et al.*, 2020). Maternal postpartum depression (PDD) is defined as a major depressive disorder episode within one year of childbirth. It is marked by symptoms such as depressed mood, loss of interest, sleep disturbances, guilt, worthlessness, and suicidal ideation lasting over two weeks (Stewart and Vigod, 2019). The risk factors for PDD may include perinatal depression and poor support from family, friends, or a partner. The prevalence of this condition is reported to be 17.8% within the first year after childbirth (Hahn-Holbrook *et al.*, 2018). First-time mothers are more likely to report depressive symptoms than those who have already had children (Bradshaw *et al.*, 2022), as their lack of experience with childbirth and childcare increases the psychosocial burden of their transition to motherhood (Ong *et al.*, 2023; Shorey *et al.*, 2015; van Roode *et al.*, 2017). A younger age at pregnancy may further increase transition challenges and the risk of postnatal depressive symptoms. Previous research on adversity during early pregnancy has primarily focused on teenage mothers; however, young adults aged 18–25 years also face unique challenges. These individuals are still in a stage of physical and psychological development, are not yet fully mature, and are subject to various psychosocial health disparities (Society for Adolescent Health and Medicine, 2017). Challenges specific to this age group include higher unemployment rates, economic instability, precarious housing conditions, and higher rates of unplanned pregnancies; this necessitates increased awareness of their vulnerability to postpartum depression compared to older first-time mothers (Harron *et al.*, 2021; Ong *et al.*, 2023; van Roode *et al.*, 2017; Zasloff *et al.*, 2007).

We defined ‘interpersonal support’ as individuals who can be genuinely relied upon when assistance is needed. Having a reliable person to turn to in times of need positively affects mental health in general adolescent and adult populations (Brugha *et al.*, 2005; Nishida *et al.*, 2024). Social support during pregnancy, especially emotional support from close individuals such as partners and family members, is crucial to reducing the risk of postpartum depressive symptoms (Cankorur *et al.*, 2015; Cho *et al.*, 2022; Faleschini *et al.*, 2019; Milgrom *et al.*, 2019; Morikawa *et al.*, 2015; Razurel *et al.*, 2017; Taylor *et al.*, 2022; Wickramaratne *et al.*, 2022). However, pregnant women sometimes experience reduced accessibility to friends and family as support (De Sousa Machado *et al.*, 2020). The number of close friends and neighbours an individual had during pregnancy had a greater impact on postpartum mental health than the quality of support (Martín-Vázquez *et al.*, 2024; Matsumura *et al.*, 2023) or satisfaction with the relationship (Morikawa *et al.*, 2015). Therefore, understanding the number of such supportive relationships is vital for designing effective support interventions. As young first-time mothers are particularly more likely to be isolated and depressed, they require robust interpersonal support beginning in early pregnancy (Henderson and Redshaw, 2016). Therefore, they should be considered a priority group for public mental health strategies to provide access to interpersonal support (Estrin *et al.*, 2019; Kim *et al.*, 2014). Identifying groups with a high need for interpersonal support and who are likely to experience positive changes through such support is important for the optimal use of limited resources (Nakamura *et al.*, 2020). Additionally, identifying the approaches to increase interpersonal support must establish a clear minimum threshold to ensure effective implementation, as practitioners and first-time mothers may struggle to take practical action without this clarity (Palmer, 2011). However, to our knowledge, no existing research has clarified the minimum number of interpersonal supporters associated with lower postpartum depressive symptoms.

This study aimed to examine the minimal number of interpersonal supporters during pregnancy associated with lower levels of postpartum depressive symptoms among first-time mothers.

## Methods

### Study setting

This population-based Mother–Infant/Newborn Tokyo Cohort (MINT cohort) study was commissioned by the Tokyo Metropolitan Government and conducted in four municipalities in Tokyo, Japan.

### Populations

The study recruited first-time mothers aged ≥ 16 years who submitted a notification of pregnancy to their municipal office in July 2022. Because the proportion of first-time mothers aged 16–25 years in the population was small, they were over-sampled to increase the number of subjects (November 2021–July 2022). Eligible individuals received a face-to-face explanation from a public health nurse regarding the web-based survey, which would be conducted for up to one year after delivery. Pregnant women who agreed to participate completed the consent procedure online and then proceeded to the first online survey. Inclusion criteria for the study were as follows: at least 16 years of age, first-time mothers, registered residents in the target municipality with no immediate plans to move out, pregnancy at earlier than 30 weeks gestation, and the ability to understand the Japanese language. During the period, 461 pregnant women who had registered their pregnancies in the target municipalities were eligible for this study. All 461 women were approached through face-to-face interactions, and 11 declined to participate, resulting in 441 participants (95.7%). Of the 441 participants, 12 did not report their age in the surveys. After excluding these 12 participants, 429 pregnant women (97.3% of those who consented) were included in the analysis.

### Data collection procedure

This study of first-time mothers comprised five waves of web-based surveys conducted at different stages: the first at the time of pregnancy notification (Wave 1, W1), the second at 20 weeks gestation (W2), the third at 1-month postpartum (W3), the fourth at 6 months postpartum (W4), and the fifth at 12 months postpartum (W5). During pregnancy (W1 and W2), data were collected on maternal demographics, mental health (using the World Health Organization-Five Well-Being Index [WHO-5] Well-Being Index), self-esteem, and social support (measured with the SSQ6). In the postpartum period (W3, W4, and W5), the surveys were expanded to include additional topics such as childbirth-related information, child health, mother–child attachment, and postpartum depression assessed using the Edinburgh Postnatal Depression Scale (EPDS), alongside items measured during pregnancy. The present analysis used data from the first (W1) and the third (W3) surveys, as postpartum depression most commonly occurs within the first 3 months after childbirth (Hutchens and Kearney, 2020; Iwata *et al.*, 2016). Additionally, we stratified the sample into two age groups—25 years and young adults aged 18–25 years. Previous research on adversity during early pregnancy has primarily focused on teenage mothers; however, young adults aged 18–25 years also face unique challenges (Aitken *et al.*, 2016). These individuals are still in a stage of physical and psychological development in extended adolescence (Sawyer *et al.*, 2018), are not yet fully mature, and are subject to various psychosocial health disparities (Society for Adolescent Health and Medicine, 2017).

## Measurements

### Maternal postpartum depressive symptoms

Postpartum depressive symptoms were assessed using the Japanese version of the EPDS (Okano *et al.*, 1996). The EPDS was developed to assess postpartum depressive symptoms after childbirth (Cox *et al.*, 1987). It is a 10-item scale that assesses a woman’s mood during the past week, with each item scored on a four-point Likert scale ranging from 0 to 3. A total score ranging from 0 to 30 was calculated, with higher scores indicating more severe depressive symptoms. The Japanese version of the EPDS has satisfactory psychometric properties, including good internal consistency, with a cutoff score of ≥ 9 indicating a diagnosis of depression (Cronbach’s alpha = 0.78; Okano *et al.*, 1996). For our analysis, we used the EPDS as a continuous variable rather than a binary variable based on the cutoff point. While the EPDS can also be used during pregnancy (Park and Kim, 2023), the WHO-5 Well-Being Index, which consists of positively framed questions, was used instead at W1 to minimise the invasiveness of the initial survey.

### Interpersonal support in pregnancy

The number of interpersonal supporters in early pregnancy (at approximately 20 weeks gestation) was measured using the first item of the Japanese version of the Social Support Questionnaire, ‘Whom can you really count on to be dependable when you need help?’ and had seven answer options: 1. ‘None’, 2. ‘One’, 3. ‘Two’, 4. ‘Three’, 5. ‘Four’, 6. ‘Five’, 7. ‘Six or more’ (Furukawa *et al.*, 1999; Sarason, Levine, *et al.*, 1983; Sarason *et al.*, 1987). In this study, our objective was to estimate the number of interpersonal supporters associated with lower postpartum depressive symptoms in first time mothers rather than to use a composite measure of social support. Therefore, we selected a single item from the scale that was the most closely aligned with the questions used in previous studies suggesting the relationship between better mental health and greater number of supporters (Brugha *et al.*, 2005; Nishida *et al.*, 2024). Given that the correlation between the first item and the summary score of the remaining five items in our survey was 0.67, we assumed that this first item preserved consistency with the scale. A prior study found that Japanese pregnant women had an average of 4.6 interpersonal supporters (Morikawa *et al.*, 2015).

### Covariates

Depressive symptoms during pregnancy were assessed using the Japanese version of the WHO-5 (Awata *et al.*, 2007; Topp *et al.*, 2015). Symptoms during pregnancy have often been reported as a critical risk factor for postpartum depression (Cruise *et al.*, 2018; Howard *et al.*, 2014) and were included as a covariate in the analysis.

### Statistical analysis

We compared demographic characteristics, depressive symptoms, and the number of interpersonal supporters between younger mothers (≤ 25 years) and older mothers (≥ 26 years). We conducted chi-square tests for categorical variables (educational attainment, marital status, employment status, prenatal and postnatal number of interpersonal supporters, prevalences of cases exceeding cutoff for depression [EPDS scores ≥ 9]) and t-tests for continuous variables (age at the first survey and prenatal and postnatal depressive symptom scores). We conducted a segment regression analysis to test whether there was a threshold at which the strength of the association between the number of interpersonal supporters and postpartum depressive symptoms changed. First, we conducted the analysis with the total sample without covariates. Second, we performed the analysis stratified by the younger and older age groups. We then repeated the analyses, adjusting for prenatal depressive symptoms, with the total sample and stratified by the age groups. The significance level was set at α = 0.05. These analyses were conducted using R version 4.4.0 with the segmented package.

## Results

### Descriptive statistics

A total of 429 first-time mothers from four municipalities in Tokyo participated in this study. The mean age was 29.0 years (SD = 5.7). The proportion of pregnant women with an education degree of less than a high school education (29.7%) and living alone (7.6%) was higher among the younger than older pregnant women (Table 1). The mean antenatal WHO-5 score was 15.2 (SD = 4.9), with no significant difference between younger and older pregnant women (*P* = 0.416; Table 2). The mean postpartum EPDS score was 6.2 (SD = 4.7), with 26.4% of pregnant women showing possible PDD. These findings are largely consistent with previous research, which reported a prevalence of 22.5% (Nakamura *et al.*, 2020). There was no significant difference in postnatal depressive symptom rates between young and older pregnant women (*P* = 0.648; Table 2). The average number of interpersonal supporters was 3.9 (SD = 1.6) during pregnancy and 3.7 (SD = 1.6) postpartum (Table 2), indicating a significant difference between the prenatal and postnatal periods (paired *t*-test: *P* = 0.005).

**Table 1.** Demographic characteristics of participants (*N* = 429)

Variable	All	Young mothers (≤ 25 years)	Older mothers (≥ 26 years)	*P-*value^a^
*N* (%)	429	158 (36.6)	271 (63.4)	
Age: mean (SD)	29.0 (5.7)	23.2 (1.8)	32.4 (4.3)	<0.001
Educational attainment				
Junior high school or high school, *n* (%)	71 (16.6)	47 (29.7)	24 (8.9)	<0.001
Further or higher education, *n* (%)	358 (83.4)	111 (70.3)	247 (91.1)	
Marital status				
Unmarried, *n* (%)	69 (16.1)	54 (34.2)	15 (5.5)	<0.001
Employment status				
Unemployed, *n* (%)	79 (18.4)	39 (24.7)	40 (14.8)	0.011

a *P-*values derived from the chi-square or *t*-test.

**Table 2.** Number of interpersonal supporters during the prenatal period, prenatal depressive symptoms, and postnatal depressive symptoms (*N* = 429)

Variable	All	Young mothers (≤ 25 years)	Older mothers (≥ 26 years)	*p* value^a^
Prenatal number of interpersonal supporters^b^	*N* = 429	*n* = 158	*n* = 271	
Mean (SD)	3.9 (1.6)	3.83 (1.6)	3.93 (1.6)	0.548
0 *n* (%)	1.0 (0.2)	1 (0.6)	0 (0.0)	0.827
1 *n* (%)	26 (6.1)	10 (6.3)	16 (5.9)	
2 *n* (%)	70 (16.3)	29 (18.4)	41 (15.1)	
3 *n* (%)	97 (22.6)	34 (21.5)	63 (23.2)	
4 *n* (%)	60 (14.0)	20 (12.7)	40 (14.8)	
5 *n* (%)	78 (18.2)	29 (18.4)	49 (18.1)	
≥ 6 *n* (%)	97 (22.6)	35 (22.9)	62 (22.9)	
Postnatal number of interpersonal supporters^b^ (*n* = 356)	*n* = 356	*n* = 129	*n* = 227	
Mean (SD)	3.7(1.6)	3.7(1.6)	3.7(1.6)	0.932
0 n (%)	2 (0.5)	0(0.0)	2(0.9)	0.693
1 *n* (%)	25(5.8)	11(8.5)	14(6.2)	
2 *n* (%)	69(16.1)	28(21.7)	41(18.1)	
3 *n* (%)	77(17.9)	23(17.8)	54(23.8)	
4 *n* (%)	55(12.8)	19(14.7)	36(15.9)	
5 *n* (%)	65(15.2)	24(18.6)	41(18.1)	
≥ 6 *n* (%)	63(14.7)	24(18.6)	39(17.2)	
Prenatal depression mean total score (SD)^c^	15.2 (4.9)	15.5 (5.3)	15.1 (4.6)	0.416
Postpartum EPDS (*n* = 352) mean total score (SD)^d^	6.2 (4.7)	5.8 (4.4)	6.4 (4.8)	0.246
Exceeding cutoff for depression (9 +), *n* (%)	93(26.4)	32(25.0)	61(27.2)	0.648

a *P-*values derived from the chi-square or *t*-test.

b Interpersonal social support was measured using the first item of the Japanese version of the Social Support Questionnaire: ‘Whom can you really count on to be dependable when you need help?’

c Depression was measured using the five-item version of the World Health Organization Mental Well-Being Index.

d EPDS = Edinburgh Postnatal Depression Scale.

### Segmented regression analysis

**Figure 1.**
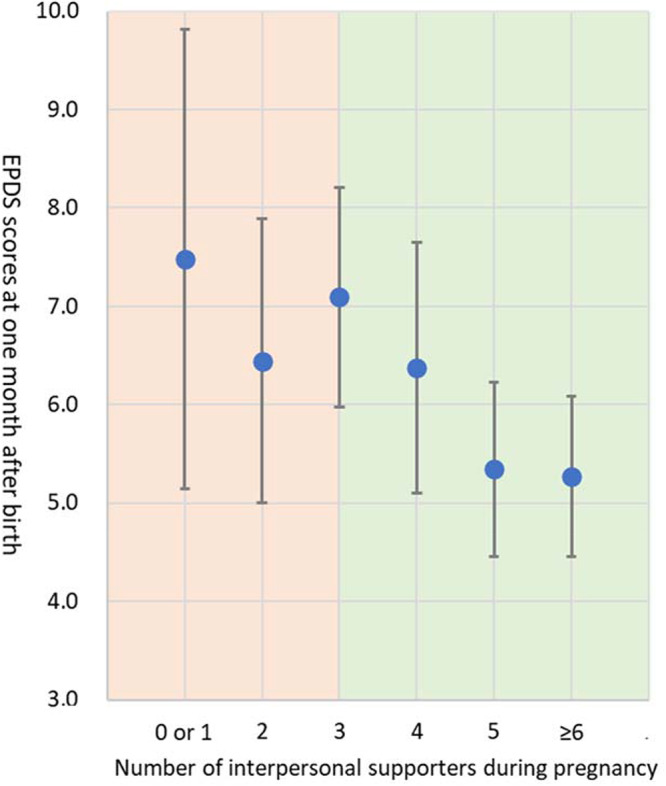
Association between the number of maternal interpersonal supporters during pregnancy and postpartum depressive symptoms 1 month after childbirth.

**Figure 2.**
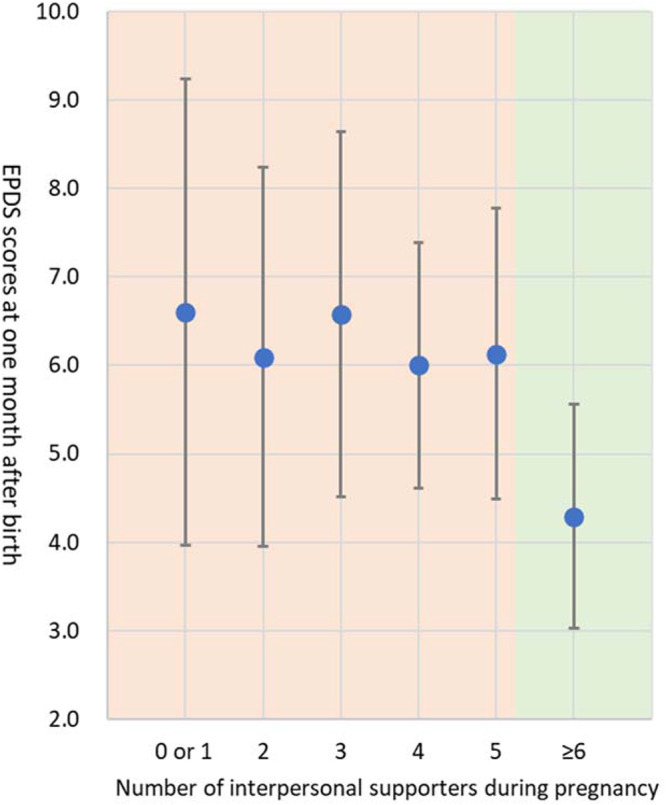
Association between the number of maternal interpersonal supporters during pregnancy and postpartum depressive symptoms one month after childbirth among young mothers ≤ 25 years-old.

**Figure 3.**
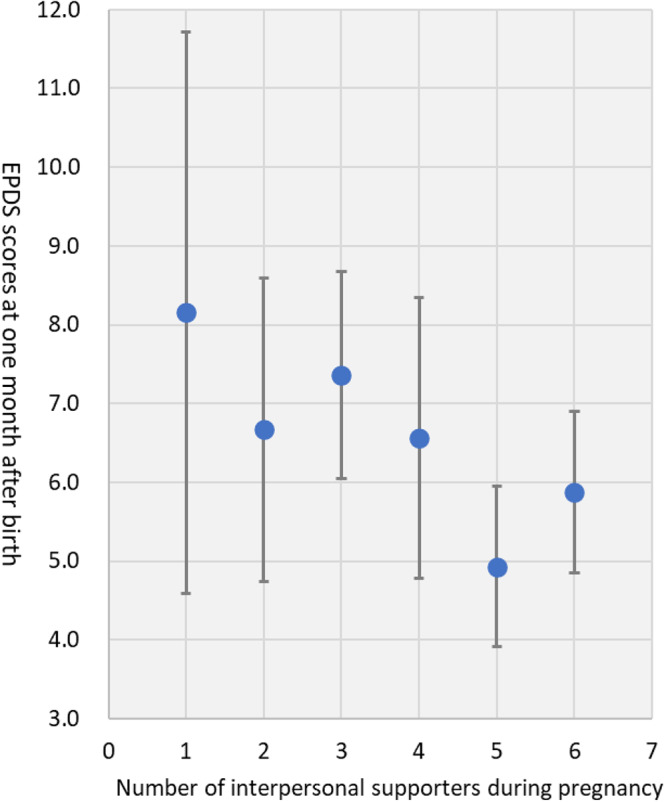
Association between the number of maternal interpersonal supporters during pregnancy and postpartum depressive symptoms 1 month after childbirth among mothers ≥ 26 years-old.

Figure 1 shows that in the segmented regression analysis, the regression line remained stable around 6.9 points for the EPDS score (range 0–30) for zero to three interpersonal supporters (β = − 0.035, *P* = 0.976), while it significantly decreased from 6.9 points to 5.6 points when the number of interpersonal supporters exceeded three (β = − 0.590, *P* = 0.013). Those with three or fewer interpersonal supporters showed higher EPDS scores than those with four or more interpersonal supporters (means of total EPDS scores: 6.92 vs. 5.58, respectively; *P* = 0.007). The results showed that the breakpoint at which the strength of the association between the number of interpersonal supporters during pregnancy and postpartum depressive symptoms changed was 3.0 (Fig. 1). These association patterns were similar even after adjusting for depressive symptoms during pregnancy (breakpoint = 3.0; the regression coefficients when the number of interpersonal supporters ≤ 3.0: β = − 0.007, *P* = .995, the number of interpersonal supporters > 3.0: β = − 0.516, *P* = 0.029). Additionally, the segmented regression analysis results among young mothers aged ≤ 25 years showed a breakpoint of 5.3 (Fig. 2). First-time mothers with five or fewer interpersonal supporters showed higher EPDS scores than those with six or more interpersonal supporters (means of total EPDS scores: 6.3 vs. 4.3, respectively; *P*= 0.029). In contrast, a breakpoint was not found among mothers aged ≥ 26 years (Fig. 3). These association patterns were similar even after adjusting for depressive symptoms during pregnancy.

Dots: Edinburgh Postnatal Depression Scale (EPDS) score one month after birth by number of interpersonal supporters during pregnancy; Orange background: numbers of interpersonal supporters less than a break point (number of interpersonal supporters = 3.0) from segmented regression analysis; Green background: numbers of interpersonal supporters more than a break point from segmented regression analysis.

Dots: Edinburgh Postnatal Depression Scale (EPDS) score one month after birth by number of interpersonal supporters during pregnancy; Orange background: numbers of interpersonal supporters less than a break point (number of interpersonal supporters = 5.3) from segmented regression analysis; Green background: number of interpersonal supporters more than a break point from segmented regression analysis.

Dots: Edinburgh Postnatal Depression Scale (EPDS) score one month after birth by number of interpersonal supporters during pregnancy.

## Discussion

This study explored the minimal number of interpersonal supporters in early pregnancy that was associated with lower postpartum depressive symptoms in a cohort of first-time mothers recruited from four municipalities in Tokyo. The results demonstrated that within the range of having zero to three supportive individuals during early pregnancy, the number of supporters was not associated with postpartum depression symptoms. However, when the number of supportive individuals was four or more, it was noted that higher numbers were associated with lower postpartum depression symptoms. Age-stratified analysis showed a linear relationship among first-time mothers aged ≥ 26 years, with the number of supportive individuals negatively associated with postpartum depressive symptoms. Conversely, among first-time mothers aged ≤ 25 years, the effects on postpartum depressive symptoms were non-significant unless they had six or more supportive individuals during early pregnancy. These findings remained consistent even after adjusting for antenatal depression.

Our findings resonate with previous studies highlighting the protective role of social support in reducing postpartum depression (Morikawa *et al.*, 2015; Taylor *et al.*, 2022). Furthermore, this study extends the literature and addresses a key gap in evidence by quantifying a minimum number of interpersonal supporters associated with lower depressive symptoms. Previous research focused on the quality of support (Cankorur *et al.*, 2015; Razurel *et al.*, 2017); our findings suggest that, at least during pregnancy, the number of reliable supporters should be considered as another key element of interpersonal support associated with lower postpartum depression.

Additionally, our study suggests that the minimum number of interpersonal supporters during pregnancy may differ according to the age at first-time pregnancy. A younger age (≤ 25 years) was associated with a need for two additional interpersonal supporters to be associated with lower postpartum depressive symptoms. Notably, many participants reported fewer supporters than the suggested threshold: only 54.8% of all first-time mothers and 22.9% of young first-time mothers met the threshold (see Table 2). Furthermore, our findings indicate that the number of interpersonal supporters reported in our study was lower than in a previous study that included multiparous women and non-nuclear families (Morikawa *et al.*, 2015). The number of interpersonal supporters declined from pregnancy to postpartum assessment, suggesting that first-time mothers may be at greater risk of social isolation during pregnancy and after childbirth (Adlington *et al.*, 2023; Seymour-Smith *et al.*, 2021). Identifying individuals with particularly low interpersonal support in early pregnancy may help enhance mental health support for mothers postpartum.

Contrary to the findings of previous studies (Harron *et al.*, 2021), we found that the mean depressive symptoms of first-time mothers aged ≤ 25 years were lower than those of first-time mothers aged ≥ 26 years. Young mothers may have fewer challenges during pregnancy than older mothers due to being physically fit and having greater flexibility in working arrangements in the early career stages. Therefore, the risk of postpartum depressive symptoms among young mothers may reflect their complex underlying needs, which necessitate intensive resource investment, interpersonal support, and multifaceted life support, including financial, educational, and career development support.

This study had several limitations. First, we used the number of trusted persons in early pregnancy to investigate the minimum number of interpersonal supporters associated with lower postpartum depressive symptoms. Previous studies have shown that sources of support influence maternal mental health, and the perceived benefit of interpersonal support depends on the social contexts of pregnant women and their families (Wickramaratne *et al.*, 2022). We have not addressed these aspects in this study. However, to our knowledge, this longitudinal study with segmented regression analysis is the first to examine the minimum number of interpersonal supporters associated with lower postpartum depressive symptoms. Second, the data employed in the present study were derived from self-report questionnaires, which may have resulted in an underestimation of depressive symptoms. Third, our data focused on urban-dwelling first-time mothers. Future studies need to be conducted to expand the target group to include multiple pregnancies and rural dwellers. Therefore, the representativeness of our findings should be interpreted with caution. Fourth, the number of subjects in this study is relatively small; thus, caution should be exercised when generalising the results.

## Conclusion

Our results suggest that having three or more interpersonal supporters in early pregnancy is associated with lower postpartum depressive symptoms among first-time mothers. Additionally, having six or more supporters appears to be associated with decreased postpartum depressive symptoms among young mothers. These findings suggest that tailored strategies to increase supporters around first-time pregnant women might be practical, depending on their age. Future research should consider expanding the sample size and conducting intervention studies to further explore the association between the number of interpersonal supporters during pregnancy and postpartum depression.

## Data Availability

The datasets generated during and/or analysed in the current study are available from the corresponding author on reasonable request.
